# The Biological Properties of the Essential Oil from the Jordan Accession of *Phagnalon sinaicum* Bornm. & Kneuck.

**DOI:** 10.3390/plants12234007

**Published:** 2023-11-28

**Authors:** Natale Badalamenti, Michela Di Napoli, Giusy Castagliuolo, Mario Varcamonti, Maurizio Bruno, Anna Zanfardino

**Affiliations:** 1 Department of Biological, Chemical and Pharmaceutical Sciences and Technologies (STEBICEF), University of Palermo, Viale delle Scienze, 90128 Palermo, Italy; natale.badalamenti@unipa.it; 2 NBFC, National Biodiversity Future Center, 90133 Palermo, Italy; 3 Department of Biology, University of Naples “Federico II”, Via Cinthia 6, 80100 Naples, Italy; michela.dinapoli@unina.it (M.D.N.); giusy.castagliuolo@unina.it (G.C.); varcamon@unina.it (M.V.); anna.zanfardino@unina.it (A.Z.); 4 Centro Interdipartimentale di Ricerca “Riutilizzo Bio-Based Degli Scarti da Matrici Agroalimentari” (RIVIVE), Università di Palermo, 90127 Palermo, Italy

**Keywords:** *Phagnalon sinaicum* Bornm. & Kneuck., Asteraceae, santolin alcohol, *α*-thujone, antimicrobials, cytotoxic activity

## Abstract

The genus *Phagnalon* Cass. (Asteraceae) is composed of widely distributed species and most of them, due to the medicinal properties they possess, are widely used in folk medicine but also as spices in the culinary field. The polar and non-polar extracts, as well as the complex mixtures of their essential oils, from several *Phagnalon* species and ssp., have shown antibiotic, antiviral, cytotoxic, and several other biological properties. In this work, the chemical composition and the antimicrobial, cytotoxic, and antioxidant properties of the Jordan accession of *Phagnalon sinaicum* Bornm. & Kneuck. essential oil (EO), an extremely rare plant that grows in ravines in the Middle East, were investigated. The EO, analyzed by GC-MS, was found to be rich in terpenoid compounds, and, in particular, in oxygenated monoterpenes, with the main compound being artemisia ketone (22.3%), followed by *α*-thujone (17.7%), and santolin alcohol (14.8%). The EO had good antimicrobial activity, especially against *Escherichia coli* Gram-negative bacterium (3 mg/mL MIC values) and was also effective in counteracting in vitro biofilm formation. Furthermore, this EO showed low cytotoxicity against immortalized human keratinocytes lines, but had good antioxidant activity on the same eukaryotic cellular models.

## 1. Introduction

The genus *Phagnalon* Cass. is a genus including thirty-five species and subspecies accepted by the POWO database [1], and almost all the suffruticose chamaephytes distributed in the main regions of the Mediterranean, and is persistently present in Iran, Turkey, and Syria. Numerous studies have been carried out on this genus of plants, which include ethnobotanical, medicinal, and phytochemical investigations, with the extraction, characterization, and individualization of the biological properties of its secondary metabolites. In fact, many works reported the medicinal use of different *Phagnalon* sp., highlighting how the use of the plants in the form of an infusion has shown anesthetic, anti-asthma, anti-inflammatory, and pain-relieving properties, and many of these studies reported the widespread use of these plants in the Palestinian region [2]. Furthermore, once dried, the leaves are used to eliminate kidney stones, possible infections [3], depressive disorders, and malfunctions of the anti-inflammatory system in the Jordan region [4,5,6]. In several Spanish areas, for example, *P. saxatile* (L.), is used in various applications: as an analgesic, anti-hypertensive, or to lower blood cholesterol levels [7,8], and, instead, a *P. sordidum* (L.) Reichenb infusion, in the Balearic Islands, is used to inhibit and to prevent the formation of kidney stones [9].

A recent review on the *Phagnalon* genus has highlighted the phytochemical characteristics of the plants investigated to date, revealing how both the extracts of the roots and those of the aerial parts contained several important metabolites such as flavonoids, benzofuran derivatives, hydroquinones, and derivatives of cinnamic acid [10]. Various biological properties have been reported in the extracts of different *Phagnalon* ssp. The polar extracts, obtained from ethanol, methanol, or hydroalcoholic mixtures of *Phagnalon sinaicum* Bornm. & Kneuck., collected in the Sinai region (Egypt), were evaluated through in vitro and in vivo tests for their antifungal potential against *Phytophthora infestans*, one of the major problems that was found in tomato crops. The results obtained showed how the use of this plant extract was able to inhibit and break down this oomycete and could be effective and widely used for the fight against *Phytophthora* in organic crops through preventing the practice of different chemical methods [11]. Further studies carried out on *P. sinaicum*, in this case, on the aqueous extract alone, showed promising antifungal activities against *Aspergillus flavus*, *A. niger*, *Curvularia lunata*, *Fusarium moniliforme*, and *Penicillium chrysogenum* [12], and moderate actions against *Botrytis fabae*, *Fusarium oxysporum*, and *P. italicum* [13]. 

Antibiotic resistance is a serious and present-day problem. An alternative to conventional antibiotics could be the use of essential oils (EOs), to gain control over antibiotic-resistant microorganisms. EOs are liquid, volatile, natural, and complex mixtures of low-molecular-weight compounds and are formed by aromatic plants as secondary metabolites, which are naturally synthesized by plants in response to attacks perpetrated by insects, herbivores, and other organisms. The presence of different compounds in the EO makes it possible to use it as an antimicrobial agent that has a low risk of developing microbial resistance [14]. The interest in natural antimicrobial products has increased in recent years. The most important and well-studied compounds come from plants, which exhibit many medicinal and antimicrobial properties, including potential activity against biofilm formation. This phenomenon causes the failure of some antimicrobial agents, and 65–80% of infections may be due to the formation of biofilms. For this reason, the EOs can be used as natural antibiofilm agents [15]. Also, the biofilm-inhibiting effect of plant extracts (extracts and EOs) has been reported against different microorganisms: *Escherichia coli*, *Listeria monocytogenes*, *Staphylococcus aureus*, and *Candida albicans* [16]. 

*P. sinaicum* Bornm. and Kneuck., a species belonging to the Asteraceae family, is a plant with a restricted geographical distribution, is rare, and spreads in Arabia, Palestine, the Egyptian region of Sinai, Yemen, Israel, and Jordan. It occurs essentially spontaneously in desert regions or in dry scrub. It is woody, perennial with a height around 15–25 cm. The leaves and branches, silvery green, are dotted with small, rounded glands at the ends. The leaves, linear-lanceolate, have an amplexicaular base and an acute to acuminate apex. The flower heads, 12–15 mm in diameter, are multi-flowered. This species can be easily distinguished from other *Phagnalon* species by the presence of glandular branches and stems [17].

There is only one literary publication, from 1994, that carried out phytochemical investigations on *P. sinaicum* [18]. It reports the presence, in the aerial parts, of metabolites such as thymol, some of its derivatives, *γ*-dienyl acetate, phytol, and squalene, all compounds structurally confirmed by spectrophotometric and spectroscopic methods. However, there is no scientific publication regarding the analysis of the EO of Jordanian accessions. Only six investigations on the compositions of *Phagnalon* ssp. EOs are reported, and their diversity will be discussed later. Continuing our studies aimed at investigating the chemotaxonomic diversity of *Phagnalon* Mediterranean species [19], the chemical and biological findings of a Jordanian accession of *P. sinaicum* Bornm. and Kneuck. EO (**PSEO**) are reported here.

## 2. Results and Discussion

### 2.1. GC and GC-MS Analysis

From the extraction process, a straw-yellow essential oil (**PSEO**) was obtained. In total, twenty-two compounds were identified from the GC-MS analysis, representing 97.3% of the total composition, as reported in Table 1. All the compounds are listed according to the calculation of the linear retention indices (LRIs) on the DB-5 MS column and to the comparisons of LRIs present in the literature. The most abundant class was that of the oxygenated monoterpenes, representing 61.7% of the total, with *β*-thujone (13.8%) and santolina alcohol (21.4%) as the majority of the compounds of the aforementioned class. The second richest class was that of the sesquiterpene hydrocarbons (28.4%), with *β*-sesquiphellandrene (16.9%) being the main metabolite of this class. Limited and reduced quantities, however, were found for the oxygenated sesquiterpenes (4.0%) and monoterpene hydrocarbons (2.2%), together with the class of other compounds (1.0%) (Table 1).

The **PSEO** differed greatly from the EO composition of *P. rupestre* ssp. *illyrian*, of *P. saxatile* var. *viride*, and of the sample obtained from the Sicilian *P. graecum* species [19]. In fact, all these EOs were found to be totally free of oxygenated monoterpenoids and very rich in monoterpene hydrocarbons. Moderate amounts of oxygenated compounds, especially monoterpenes, were found in the EOs extracted from two different accessions of *P. sordidum*, one Algerian [20] and the other French (Corsica) [21], but the majority of the **PSEO** compounds (*α*-thujone, santolin alcohol, etc.) were not detected. Among the sesquiterpene hydrocarbons, *β*-sesquiphellandrene has never been detected in any other *Phagnalon* accession and, in this case, germacrene D, present in high quantities in the Turkish species of *P. graecum* (21.30%) [22] and *P. sordidum* (3.12–7.65%) [20,21], was totally absent. The compounds identified, like artemisia ketone and santolina alcohol, are biogenetically irregular derivatives and have been identified in several species of the genus *Artemisia* and *Santolina* [23], *Achillea* [24], and *Ericephalus* [25]. These two compounds were both identified only in one EO of the *Phagnalon* species, collected in the state of Israel [26] and were very similar qualitatively to **PSEO**.

### 2.2. Antimicrobial Activity of PSEO

**Figure 1:**
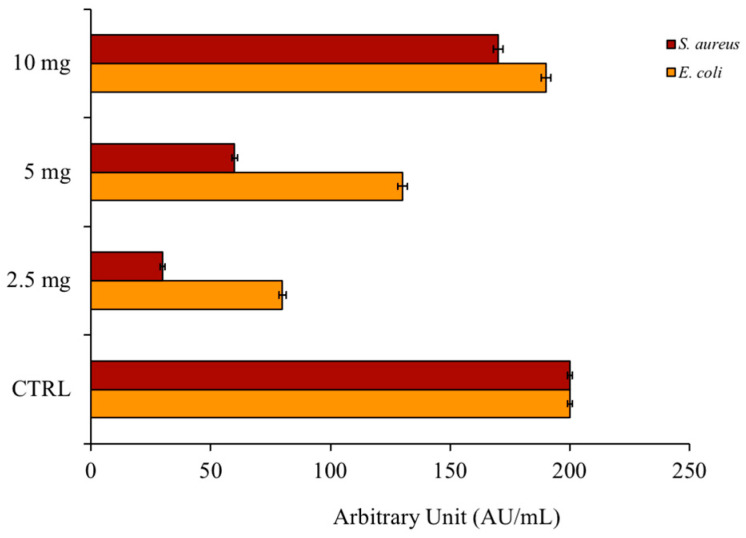
Inhibition of bacterial growth. The image shows the inhibition halos, expressed in AU/mL, of **PSEO** against *E. coli* and *S. aureus*. The positive control was ampicillin and the negative control was DMSO.

The first experimental approach to verify the antimicrobial activity of a new compound is the Kirby and Bauer assay. It is a qualitative method that quickly returns information on the inhibition of the microorganism growth around a compound-tested drop. The antimicrobial activity of the **PSEO** was tested against a Gram-negative *Escherichia coli* and a Gram-positive *Staphylococcus aureus* bacterial model. Figure 1 shows that the resulting inhibition zone on the plate grows as the quantity of EO increases. As can also be seen from the figure, the antimicrobial activity seems to be directed more towards Gram-negative bacteria (orange bars).

**Figure 2:**
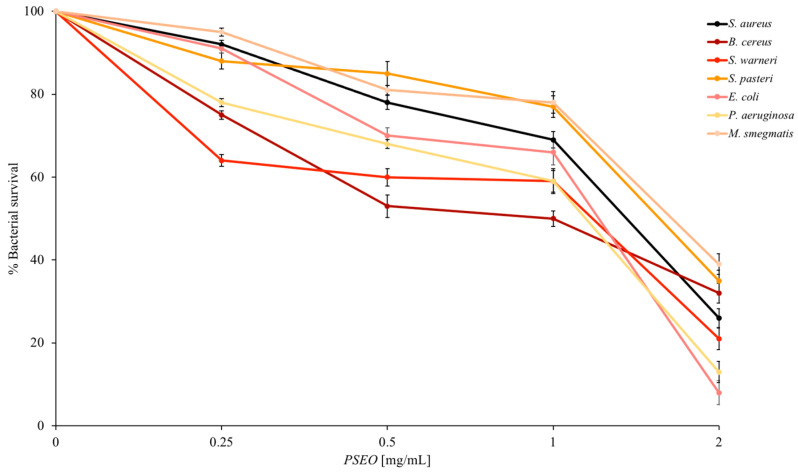
Antimicrobial activity against Gram-positive and Gram-negative strains shown with dose–response curves. The tests were performed in three independent experiments. Standard deviations are always less than 5%.

The construction of the dose–response curves (Figure 2) made it possible to validate the data in a more quantitative way. The indicator strains set has been expanded, using different bacteria that establish interactions with human cells. These are both Gram-negative and -positive bacteria, including bacterial strains in oral, gastro-intestinal, and skin infections. The proportionality between the quantity of **PSEO** administered to the bacteria and the decrease in their cell survival remains stable at relatively low concentrations for a mixture of substances such as EO (2 mg/mL). The **PSEO** is particularly effective against *E. coli* or *P. aeruginosa*, which have a very low survival rate (around 10%). Furthermore, these data are in agreement with the qualitative data, in which **PSEO** is more active against Gram-negative bacteria.

To complete the antimicrobial activity analysis, a minimum inhibitory concentration (MIC) test for microbial growth was performed against all indicator strains. As shown in Table 2, the lowest MIC values are obtained for the Gram-negative strains, in agreement with the data previously shown.

**Figure 3:**
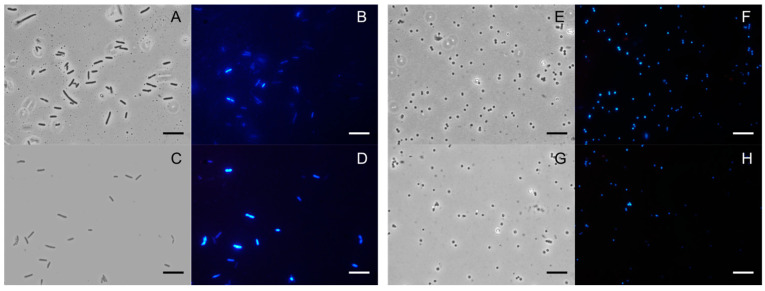
Analysis with fluorescence microscopy. Panels show *E. coli* bacterial cells (**A**–**D**) and *S. aureus* bacterial cells (**E**–**H**). Panels (**A**,**C**,**E**,**G**) show the cells observed under the optical microscope, and panels (**B**,**D**,**F**,**H**) show the cells observed under the fluorescence microscope. Untreated bacterial cells (**A**,**B**,**E**,**F**); cells treated with **PSEO** (**C**,**D**,**G**,**H**). Scale bars: 1 µm (**A**–**H**).

To find information on the possible antimicrobial target, fluorescence microscopy experiments were performed. As shown in Figure 3, no damaged membranes are observed after incubation with the **PSEO** at a concentration of 2 mg/mL (for negative strain) and 5 mg/mL (for positive strain) for approximately 4 h. Two specific DNA dyes were added to the control and to the sample also containing the EO: DAPI, which gave blue fluorescence and passed through intact membranes, and propidium iodide (PI), which gave red fluorescence and only passed through damaged membranes. Both the Gram-negative (panels A–D) and Gram-positive bacteria (panels E–H) did not show red fluorescence and gave similar results to the controls (B and F). This result highlighted that the EO target did not appear to be bacterial membranes. Our group has been engaged for years in researching new natural antimicrobial substances, compared to other EOs such as thyme, fennel, orchid, and others [27]. In fact, there are many mechanisms of action implemented given the EO’s components [28]. Although many EOs target membranes, some compounds and mixtures of compounds can affect growth regulation, nutritional balances, and energy conversion in bacteria [29].

### 2.3. Antibiofilm Activity of PSEO

**Figure 4:**
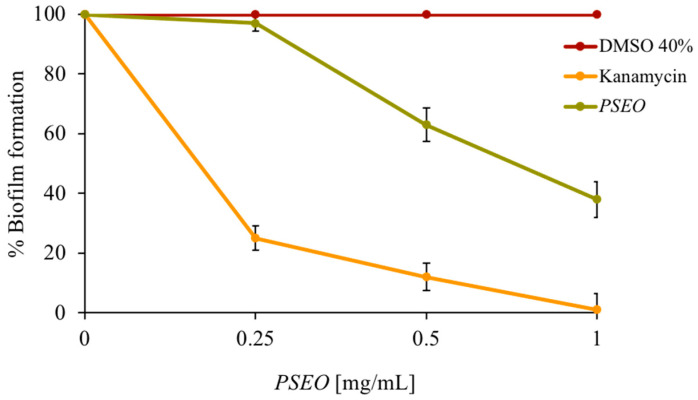
Antibiofilm activity. The percent inhibition of *M. smegmatis* biofilm is shown in the figure. Different concentrations of **PSEO** were tested (*x*-axis). The tests were performed in three independent experiments. Standard deviations are always less than 5%.

Demonstrating the effectiveness of a new substance only on planktonic cells is a good starting point, but implementing the research at the level of a complicated microbial organization, such as the biofilm, is necessary. The ability to form a biofilm is a significant concern, and a biofilm’s resistance is supported by genetic, physical, and physiological mechanisms [30]. In biofilms, cells are more resistant to disinfectants and antibiotics, and are also more protected and increase their genetic variability [31]. *Mycobacterium smegmatis* was used as a model organism in the biofilm development study. After analyzing the dose–response curves obtained with this strain by progressively increasing the amount of EO, the concentrations that did not cause the death of the microorganism were chosen for the biofilm inhibition experiments. If the planktonic cells die, it is clear that they cannot produce biofilm, standardizing the experiment for the number of cells added and considering the biofilm formation of a control (no **PSEO** added). In Figure 4, it is noted that the **PSEO** inhibits biofilm formation by 60%. DMSO was used as a negative control and the antibiotic kanamycin as a positive control, which gives the total biofilm inhibition at 1 mg/mL. The good antibiofilm activity of the **PSEO** made it a good candidate in various sectors, ranging from oral hygiene to nutraceutical and cosmetic use. However, in order to pursue this kind of application, in vivo biofilm studies need to be carried out.

### 2.4. Antioxidant Activity of PSEO

For nutraceutical and cosmetic companies, an important property of a substance is its antioxidant power. In vitro tests were carried out, verifying the ability to screen oxygen radicals, as can be seen in Table 3. The **PSEO** is very effective at screening hydrogen peroxide: even at very low concentrations, there is a 50% inhibition of ROS (around 0.025 mg/mL). In the same table, we can see how the scavenging activity against ABTS is less marked, with an IC_50_ of approximately 2 mg/mL. 

**Figure 5:**
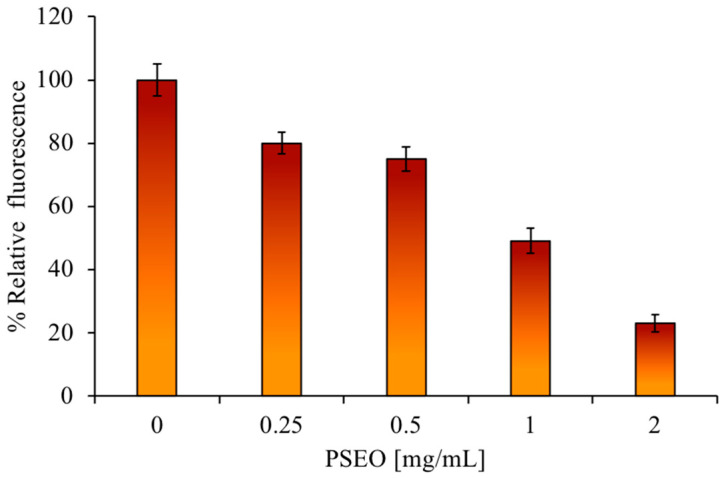
Effects of **PSEO** on intracellular ROS generation in HaCaT cells upon H_2_O_2_ treatment.

Before testing the antioxidant activity on the HaCat cell line, it was necessary to carry out a cytotoxicity assay using the **PSEO** at different concentrations up to 6 mg/mL, both at 4 h and 24 h. No cytotoxicity was observed under these experimental conditions (Figure S1). The ROS assay stain was used to confirm the obtained in vitro data about the antioxidant activity of the **PSEO**. By pre-treating the eukaryotic cells with increasing concentrations of the **PSEO**, and subsequently giving a stimulus with hydrogen peroxide, the ROS concentration decreases. As shown in Figure 5, there was a massive production of ROS in the control (untreated cells), with an increase in absorbance. In the **PSEO** samples, the absorbance decreased as the EO concentration increased, confirming that the **PSEO** pretreatment significantly lowers the concentration of ROS produced by the cells following a H_2_O_2_ pulse. 

## 3. Materials and Methods

### 3.1. Plant Material Collection

The flowering aerial parts of wild *P. sinaicum* were collected, cleaned, and stored at 0 °C in Al-Jafr, a small city located in the far south of Jordan, about 200 km from the capital Amman (30°17′36″ N 36°12′50″ E), in April 2023. The collected samples were identified by Prof. Vincenzo Ilardi and one exemplary was preserved at the University of Palermo Herbarium (Voucher no. PAL 108769). 

### 3.2. Isolation of Essential Oil

The samples (≈300 g), still fresh, were chopped manually and then through a Waring mixer and finally subjected to hydrodistillation for 3 h, according to the standard procedure described in the European Pharmacopoeia [32]. The extracted EO was dried over Na_2_SO_4_ and refrigerated under N_2_ until the GC analysis. The yield obtained was 0.10% (*w*/*w*).

### 3.3. GC and GC-MS Analysis

GC-MS analysis was performed using a Shimadzu QP 2010 plus gas chromatograph equipped with an AOC-20i autoinjector (Shimadzu, Kyoto, Japan) equipped with a FID, capillary column (DB-5 MS) 30 m × 0.25 mm i.d., 0.25 μm film thickness, and a data processor. The oven program was as follows: temperature increase to 40 °C for 5 min, at a rate of 2 °C/min up to 260 °C, then isothermal for 20 min. Helium was used as carrier gas (1 mL min^−1^). The injector and detector temperatures were set to 250 °C and 290 °C, respectively. A total of 1 μL of EO solution (3% EO/hexane *v*/*v*) was injected in split mode 1.0; MS range 40–600. The settings were as follows: ionization voltage, 70 eV; electron multiplier energy, 2000 V; transfer line temperature, 295 °C; solvent delay, 3.5 min. Linear retention indices (LRIs) were determined using retention times of *n*-alkanes (C_8_–C_40_) and peaks were identified by comparison with mass spectra and comparison of their retention indices with WILEY275 libraries, NIST 17, ADAMS, and FFNSC2.

### 3.4. Bacterial Strains

The antimicrobial activity was evaluated using Gram-positive and Gram-negative strains: *Staphylococcus aureus* ATCC6538P, *Bacillus cereus* ATCC10987, *Streptococcus warneri*, *Streptococcus pasteuri*, *Escherichia coli* DH5α, *Pseudomonas aeruginosa* PAOI and *Mycobacterium smegmatis* mc^2^ 155.

### 3.5. Antimicrobial Activity Assay

The presence of antimicrobial molecules in **PSEO** was detected using the agar diffusion assay of Kirby–Bauer [33]. Masses of 2.5, 5, 10 mg of **PSEO** were placed on Luria–Bertani agar plates which were overlaid with 10 mL of soft agar (0.7%) mixed with 10 μL of strains grown for 24 h at 37 °C. The negative control was dimethylsulfoxide (DMSO) 40%, used to resuspend the EO; the positive control was represented by the antibiotic ampicillin. Plates were incubated overnight at 37 °C and the antimicrobial activity was calculated according to the equation cited by Napolitano et al. [34]. For the second experiment, samples with a final volume of 500 μL were prepared; they contained bacterial cells, extract of **PSEO** at different concentrations (0.25, 0.5, 1, and 2 mg/mL), and 20 mM pH 7.0 of NaP buffer up to final volume. Samples without extract were used as a positive control. The negative control contained bacterial cells with DMSO 40%. After 4 h of incubation at 37 °C, all samples were prepared and then plated on LB agar in Petri dishes that were finally incubated at 37 °C overnight. The following day, the surviving percent of bacterial cells was estimated by counting the number of colonies. Each experiment was performed in triplicate and the reported result was an average of three independent experiments. (*p* value was <0.05). 

### 3.6. Determination of Minimal Inhibitory Concentration

Minimal inhibitory concentrations (MICs) of PSEO against all strains were determined as described by Di Napoli et al. [35]. Following overnight incubation at 37 °C, MIC values were determined as the lowest extract concentration responsible for no visible bacterial growth.

### 3.7. DAPI/PI Dual Staining and Fluorescence Microscopy Image Acquisition

For dual staining, 100 µL of *E. coli* DH5α and *S. aureus* ATCC6538P (mid-logarithmic phase) was incubated in the dark for 4 h at 37 °C in agitation in the presence or absence of **PSEO**, at concentrations of 2 mg/mL (for *E. coli*) and 5 mg/mL (for *S. aureus*). After 4 h, 10 µL of bacterial culture was mixed with DAPI solution (40, 6-diamidino-2-phenylindole dihydrochloride; Sigma Aldrich, Milan, Italy) and PI (propidium iodide; Sigma Aldrich, Milan, Italy). Samples were observed using an Olympus BX51 fluorescence (Olympus, Tokyo, Japan) using a DAPI filter (excitation/emission: 358/461 nm) [35].

### 3.8. Antibiofilm Activity Assay

Crystal violet dye was used to evaluate the biofilm formation of *M. smegmatis* mc^2^ 155. A 24-well plate was prepared in which each well contained a final volume of 1 mL; the negative control was represented by only bacterial cells and medium, the positive control was represented by bacterial cells with antibiotic kanamycin 2 µg/mL, the other samples contained cells and **PSEO** [0.25, 0.5, 1 mg/mL]. The plate was incubated at 37 °C for 36 h. The OD of the crystal violet present in the dual staining solution was measured at 570 nm. 

### 3.9. Antioxidant Activity

#### 3.9.1. ABTS Scavenging Capacity Assay

This assay was performed according to Re et al. [36], which is based on ABTS radical cation scavenging. A total of 1 mL ABTS solution was added to 100 μL of **PSEO** (0.05–2 mg/mL concentrations). Finally, the absorbance was measured at 734 nm against a blank, and the percentage inhibition of ABTS radicals was determined from the following equation: ABTS^+^ radical scavenging activity (%) = (1 * A_S_/A_C_) × 100, where A_C_ is the absorbance of the ABTS solution and A_S_ is the absorbance of the sample at 734 nm. The concentration required for 50% inhibition was determined and represented as IC_50_. 

#### 3.9.2. Hydrogen Peroxide Scavenging Assay

The hydrogen peroxide stability was measured as the absorbance at 240 nm of 1 mL of fresh hydrogen peroxide solution (50 mM potassium phosphate buffer, pH 7.0; 0.036% (*w*/*w*) H_2_O_2_) [37]. Different concentrations of **PSEO** (0.05–2 mg/mL) were incubated at 20 °C in 1 mL of hydrogen peroxide solution. The percentage of peroxide removed was calculated as follows: peroxide removed (%) = (1 * A_S_/A_C_) × 100, where A_C_ is the absorbance of 1 mL of hydrogen peroxide solution and A_S_ is the absorbance of the sample at 240 nm.

### 3.10. Eukaryotic Cell Culture and Antioxidant Test

HaCat (human keratinocytes) cells are a spontaneously transformed aneuploid immortal keratinocyte cell line from adult human skin, widely used in scientific research. **PSEO** was added in a complete growth medium for the cytotoxicity assay [38]. HaCaT cells were plated in 12-well plates and then incubated at 37 °C with 5% CO_2_ for 24 h. Cells were treated with PSEO at different concentrations (0.25, 0.5, 1, and 2 mg/mL). After 60 min of treatment, H_2_O_2_ (800 µM) was added to each well for the next 3 h before detecting intracellular ROS. ROS Assay Stain (88-5930, Invitrogen, Waltham, MA, USA) was administered to cells in culture media according to the manufacturer’s protocol. Fluorescence intensity (530 nm) was measured using a Synergy H4 hybrid microplate reader (Agilent, Santa Clara, CA, USA).

## 4. Conclusions

In this study, for the first time, a chemical analysis of harvested *P. sinaicum* essential oil, collected in Jordan, was carried out using GC and GC-MS. The essential oil was found to be rich in terpenes with oxygenated monoterpenes as the most abundant class (61.73%), with *β*-thujone (13.77%) and santolina alcohol (21.43%) as the main compounds. *β*-Sesquiphellandrene (16.89%) was, instead, the major compound of sesquiterpene hydrocarbons class (28.39%). The *P. sinaicum* essential oil appeared to have good antimicrobial activity, especially directed against Gram-negative bacteria, in which bacterial membranes are not the primary target. Furthermore, according to some investigations, synergistic volatile compounds such as santolina alcohol and *β*-sesquiphellandrene contained in essential oils are mainly responsible for antibacterial activity. The ability of this essential oil to inhibit biofilm formation, causing oral diseases and infections on the skin, is very interesting. Furthermore, the *P. sinaicum* essential oil ROS scavenging activity is an important property for nutraceutical or cosmetic applications.

## Figures and Tables

**Table 1:** Chemical composition of Jordan accession of *Phagnalon sinaicum* (**PSEO**) essential oil.

No.	Compounds ^a^	LRI ^b^	LRI ^c^	Area ^d^ (%)
1	*α*-Pinene	931	938	0.4
2	*β*-Phellandrene	971	978	0.6
3	*β*-Pinene	982	980	0.1
4	*α*-Terpinene	1018	1022	1.1
	**Monoterpene Hydrocarbons**			**2.2**
5	Eucalyptol	1034	1040	2.1
6	Santolina alcohol	1038	1047	21.4
7	Artemisia ketone	1061	1080	13.2
8	Artemisia alcohol	1092	1100	0.2
9	*α*-Thujone	1102	1125	8.8
10	*β*-Thujone	1127	1135	13.8
11	Linalool	1110	1176	1.0
12	Terpinen-4-ol	1180	1185	0.4
13	*α*-Terpineol	1199	1199	0.8
	**Oxygenated Monoterpenes**			**61.7**
14	*α*-Copaene	1372	1371	1.0
15	*trans*-*β*-Farnesene	1455	1458	0.6
16	Germacrene D	1480	1480	5.2
17	Bicyclogermacrene	1494	1490	4.7
18	*β*-Sesquiphellandrene	1523	1531	16.9
	**Sesquiterpene Hydrocarbons**			**28.4**
19	Spathulenol	1571	1571	2.1
20	*α*-Cadinol	1653	1652	1.9
	**Oxygenated Sesquiterpenes**			**4.0**
21	Hexanal	802	808	0.3
22	2-Hexenal	832	862	0.7
	**Other Compounds**			**1.0**
	**Total Composition** (**%**)			**97.3**

^a^ Compounds are classified in order of LRIs of the apolar column (DB-5 MS); ^b^ LRIs on a DB-5 MS apolar column; ^c^ LRIs reported for DB-5 MS column reported in the literature; ^d^ area is the peak volume percentage of compound in the essential oil (EO) sample.

**Table 2:** Determination of minimum concentration (MIC) values that inhibit bacterial growth. The MIC is expressed in mg/mL of **PSEO** against Gram-positive and Gram-negative bacteria. Values were obtained from a minimum of three independent experiments.

Strains	MIC [mg/mL]
***S.*** ***aureus***	6
***S. warneri***	10
***S. pasteuri***	10
***B. cereus***	10
***E. coli***	3
***P. aeruginosa***	4
***M. smegmatis***	>10

**Table 3:** Concentration at 50% scavenging activity. ABTS: 2,20-azino-bis (3-ethyl-benzothiazoline-6-sulfonic acid); H_2_O_2_: hydrogen peroxide. The positive control is ascorbic acid for ABTS and resveratrol for H_2_O_2_.

Sample	IC_50_ of ABTS (mg/mL)	Sample	IC_50_ of H_2_O_2_ (mg/mL)
**PSEO**	2	**PSEO**	0.025
Ascorbic acid	0.00003	Resveratrol	0.00005

## Data Availability

All data and materials are available upon request from the corresponding author.

## Supplementary Materials

The following supporting information can be downloaded at https://www.mdpi.com/article/10.3390/plants12234007/s1, Figure S1: Determination of the cytotoxic activity of **PSEO**. The image shows the percentage of cell survival of the HaCat cell line treated with different concentrations (*x*-axis) of EO for 4 and 24 h. Cell survival percentage is shown on the *y*-axis. The tests were performed in three independent experiments. Standard deviations are always less than 5%.
