# Supplementary material for: The Biological Properties of the Essential Oil from the Jordan Accession of Phagnalon sinaicum Bornm. & Kneuck
Source: Plants (Basel). 2023 Nov 28;12(23):4007. doi: 10.3390/plants12234007 (PMC10708455; doi:10.3390/plants12234007)
Supplement: Supplementary file 1 [file plants-12-04007-s001.zip › plants-2727139-supplementary.pdf]

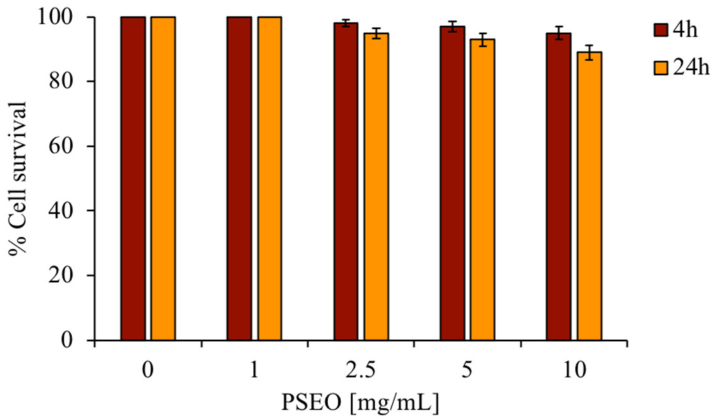

Figure S1: Determination of the cytotoxic activity of **PSEO**. The image shows the percentage of cell survival of the HaCat cell line treated with different concentrations (x-axis) of EO for 4 and 24 h. Cell survival percentage is shown on the y-axis. The tests were performed in three independent experiments. Standard deviations are always less than 5%.
